# Knowledge structure and emerging trends of cognitive impairment induced by sleep deprivation: A bibliometric analysis based on CiteSpace and VOSviewer from 2000 to 2022

**DOI:** 10.1097/MD.0000000000034776

**Published:** 2023-10-06

**Authors:** Kai Yu, Lei Hao, Fan Bu, Yuanzhi Guo, Yaqi Duan, Rui Hu, Ji Lu, Peng Li

**Affiliations:** a The First Affiliated Hospital of Inner Mongolia Medical University, Inner Mongolia, China; b Urology Department, The First Hospital of Jilin University Changchun, Changchun, China; c Department of Pathophysiology, Inner Mongolia Medical University, Inner Mongolia, China.

**Keywords:** Citespace, cognitive disorder, sleep deprivation, visual analysis

## Abstract

This paper implements a bibliometric approach to investigate the research hotspots and future research directions in the relevant field literature. It also offers research ideas and methods for preventing and treating cognitive impairment induced by sleep deprivation in the clinical setting. The evolution of various clusters in the field is summarized through Citespace’s projection function for keywords in the literature. CiteSpace and Vosviewer are utilized to analyze and visualize the attributes of the articles, including number of publications, citation frequency, country/region, institution, journal, authors, keywords, and references, from the 2280 publications obtained. A total of 2280 publications were collected, with the number of papers and citations in the field continuously increasing year by year. The most influential country in this field is the United States, and the University of Washington is the most influential institution. The most authoritative journal in the field is identified as SLEEP. Sleep deprivation, prefrontal cortex, and performance are the current topics of interest. The article with the strongest citation burst, lasting from 2015 to 2018, is “Sleep Drives Metabolite Clearance from the Adult Brain.” The most influential article and co-cited reference, “Neurocognitive Consequences of Sleep Deprivation,” highlights that sleep deprivation from various causes may lead to cognitive impairment. Future research should investigate all forms of cognitive impairment resulting from sleep deprivation. The findings of this study will assist researchers in improving their knowledge structure, identifying research hotspots, and revealing future directions in the field.

## 1. Introduction

Cognitive dysfunction refers to abnormalities in neurological functions related to the intake, storage, reorganization, and processing of information, resulting from various causes such as impaired judgment, attention, memory, reduced reasoning, executive function, and communication difficulties.^[1]^ With global aging, changes in lifestyle, and diet, the prevalence of cognitive dysfunction is gradually increasing,^[2–4]^ which seriously affects the quality of life of patients^[5]^ and increases the burden of care^[6]^ also the psychosocial distress.^[7]^ Studies have indicated that various diseases, such as Parkinson’s syndrome, Alzheimer’s disease, stroke, and diabetes, can cause cognitive dysfunction. Furthermore, age, hypertension, living quality, and oxidative stress are all risk factors for cognitive dysfunction.^[8]^ Cognitive dysfunction can be classified by severity into mild cognitive impairment (MCI or mild NCD) and severe cognitive dysfunction, MCI can progress to dementia in varying degrees,^[9]^ and some studies have shown that early intervention for MCI patients can slow down the rate of cognitive decline, therefore, early personalized clinical management of MCI patients is beneficial to improve cognitive function.^[10–12]^ There are no clinically recognized drugs that can reverse or modify the progression of MCI to dementia. Thus, it is imperative to improve research aimed at cognitive impairment from novel perspectives, rectifying misconceptions such as stigma and emphasizing the importance of early detection and treatment as effective measures to delay onset of the condition.^[13]^

Due to the COVID-19 pandemic, individuals’ lifestyle behaviors have significantly altered, resulting in heightened psychological stress and slumber disturbances. Sleep, a physiological process, is essential for regaining energy, enhancing cognitive abilities, and controlling digestion, representing a fundamental need for human survival. Proper sleep is vital in combating mental exhaustion, aiding in the restoration of physical strength and vigor, which, in turn, may enhance performance in our daily academic and occupational duties.^[14]^ Sleep deprivation (SD) is a condition of intentionally induced sleep reduction, commonly utilized in sleep studies. However, with the progress of society, SD is becoming a more prevalent social phenomenon.^[15]^ Studies conducted abroad have shown that approximately 35% of adults have a sleep duration of less than 7 hours per day. Additionally, approximately 73% of high school students get less than 8 hours of sleep at night, while 58% of secondary school students get less than 9 hours of sleep. Sleep deprivation can lead to significant and multidimensional health damage, posing a threat to various bodily systems, such as the nervous and endocrine systems,^[16,17]^ which can cause, among other things, impaired cognitive function A range of physical, mental and behavioral abnormalities, including impaired cognitive function.^[18]^ The Centers for Disease Control and Prevention recently classified SD as an epidemic in the United States, with 35% of Americans reportedly sleeping less than the recommended 7–9 hours per night. SD can impair attention and memory, interfere with normal work and learning, lead to decreased academic performance, increased work errors, and even accidents; in addition, SD can lead to impaired cognitive function and affect one’s ability to live life.^[17]^

Developing health beliefs about good sleep can help reduce the incidence of cognitive impairment.^[19]^ Studies have shown that sleep restriction or sleep deprivation can lead to cognitive decline^[20]^ and can also increase the risk of Alzheimer’s disease. Previous studies have found that chronic sleep deprivation can cause significant neurological damage, directly damaging hippocampal neurons, and therefore sleep deprivation is often associated with cognitive impairment.^[21,22]^ Potential mechanisms underlying cognitive impairment caused by sleep deprivation include oxidative stress, alterations in neurotransmitters, structural damage to the hippocampus, changes in gene expression, and inhibition of long-term potentiation. Of these, hippocampal structural damage is closely associated with learning and memory in the central nervous system and is particularly critical for cognitive function. Apoptosis and mitochondrial damage in the hippocampus may also contribute to cognitive dysfunction. Research has shown that cells in the CA1 hippocampal region become disorganized, and the niche is reduced or absent following sleep deprivation in rats. In addition, the ultrastructure of blood vessels, axons, and mitochondria in the CA1 region are abnormally altered, with dilated endoplasmic reticulum, axonal edema, and obvious mitochondrial damage, potentially impacting the rats’ ability to learn and remember.^[22]^ In addition, previous studies have found that in chronic deprivation of fast-acting eye phase sleep animal experiments, the experimental mice. In addition, previous studies have found that after sleep deprivation, the deposition of Αβ protein in the brain of mice was significantly increased and learning and memory abilities were significantly impaired, thus causing cognitive dysfunction in mice. The stress response caused by sleep deprivation, prolonged awakening time and reduced melatonin in the brain may be the cause of Αβ protein deposition due to sleep deprivation.^[23–25]^ And it has been shown that chronic sleep deprivation can lead to disruption of the melatonin system in vivo^[26]^ and several studies have shown that melatonin can alleviate the symptoms of sleep deprivation-induced cognitive dysfunction.^[16]^ Some patients with cognitive impairment may have suicidal behaviors, and we need to recognize it early and take corresponding preventive measures,^[27]^ One effective approach is to reduce to some extent the possible exposure of patients to environments that may trigger disease.^[28]^

Bibliometrics has been widely used in many life sciences such as medicine, biology, public health and so on.^[29,30]^ Bibliometrics as a new integration technology provides new ideas for exploring the research status, content and hotspots in various fields.^[31]^ By analyzing the potential information of the past, we can gain better insight into the future.^[32,33]^ This study uses bibliometrics, with the help of Excel and Citespace software, to visualize and analyze the volume of articles, authors, institutions, and keywords of sleep deprivation-related cognitive dysfunction-related research from 2000 to 2022 at home and abroad, so that our scholars can In order to provide a timely and accurate tracking of the current situation of domestic research and hot trends, and to provide a reference basis for subsequent research directions.

## 2. Materials and methods

### 2.1. Raw data acquisition

The Web of Science Core Collection database was used as the original literature data source, with sleep deprivation and cognitive dysfunction as search terms. Due to the number of cited papers and other parameters that may vary in search time, the search time for this study was specified as January 25, 2023, and the detailed search strategy was (TS = (Cognitive Dysfunctions) OR TS = (Cognitive Disorder) OR TS = (Mental Deteriorations) OR TS = (Cognitive Declines)) AND (TS = (Sleep Deprivation) OR TS = (Sleep Insufficiency) OR TS = (Insufficient Sleep Syndromes)).

The total number of relevant literature of WOS was 2334, and to ensure the accuracy and validity of the data, the relevant papers were searched for a limited period of time from January 1, 2000 to December 31, 2022, and the types of literature research included research papers, review papers and other types of papers. After screening, 2280 original literature data were obtained, and all original literature data results were downloaded and saved in the plain text format of “full record with cited references.”

### 2.2. Data analysis and related parameter settings

The data obtained from WOS were imported into Excel, Citespace and VOSviewer software to analyze and statistically describe the number of articles, the main authors, research institutions and their citations and collaborations, major published journals and their citations in the field of sleep deprivation-related cognitive dysfunction research in recent years, in order to obtain the distribution structure, volume changes and The trend of development.

In Citespace 6.1.R6 software, the time selection was set to 2000 to 2022, the time slice was selected as 1 year, and the rest of the options were kept as default settings, the number of citations or occurrences per year was 10% of the literature, and the maximum number of literature per time slice (time slicing) was 100. In the pruning algorithm, Pathfinder, Pruning sliced networks, Pruning the merged network are selected at the same time, and if there are software operation errors, etc., the default settings are kept in the pruning algorithm without Pathfinder, Pruning sliced networks, and Pruning the merged network are not checked in the pruning algorithm.

### 2.3. Analysis of visualization results

In the node type, country, institution, journal, author, keyword and highly cited literature are analyzed separately. the graph generated by Citespace consists of nodes and the corresponding lines. The size of a node corresponds to the frequency of that node in the corresponding analysis module, the color of a node corresponds to the research time of that node, and the links between nodes indicate the co-occurrence or co-citation between nodes. In keyword detection, log-likelihood rate is usually used as the main method of keyword clustering analysis, and keyword class clusters with high confidence are clustered at the clustering module value (Modularity Q) and mean silhouette value (Mean Silhouette) based on co-occurrence analysis. Further to the keyword clustering analysis, the keywords were visualized with a timeline spectrum analysis, and cognitive dysfunction due to cognitive dysfunction was analyzed by timeline spectrum.

In burst word detection, γ(0,1) is set to 0.5 and the minimum duration is set to 1. If less than 20 burst words are retrieved, the value of γ is decreased by 0.1 each time until more than 20 words are retrieved. The thickness of the link represents the tightness of the connection between 2 nodes, and the color of the link represents the duration of the connection between the nodes. The node with the outermost circle in purple usually means that its centrality is greater than 0.1, indicating that the node has significant influence in the module. The data source in VOSviewer is bibliographic data for the calculation of total citations and total connection strength, and the threshold is usually set to 5 articles in the calculation, and if no valid class clusters are formed, the threshold is gradually reduced by 1 until a more obvious clustering situation occurs. In the graph generated by VOSviewer only keep the nodes that are connected to each other, the layout is set to attract (Attraction) 2, repulsion (Repulsion) -1, and the rest of the settings remain unchanged by default settings.

## 3. Results

### 3.1. Analysis of the number of articles issued

**Figure 1.**
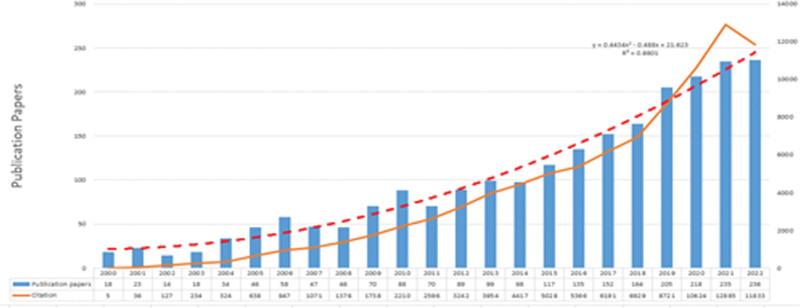
Annual changes in the number of literatures related to sleep deprivation related cognitive dysfunction in Web of Science Core Collection database between 2000 and 2022.

Of the 2280 papers queried, 1750 (76.75%) were original research papers and 471 (20.66%) were review papers. According to the data analysis of the retrieved primary literature, the number and trend of papers on sleep deprivation-related cognitive dysfunction, as shown in Figure 1. The number of papers in the related field has gradually increased since 2000. the number in 2022 was 13.1 times higher than that in 2000. The number peaked in 2022 with 236 papers. As shown by the solid line of the trend, the number of papers fluctuates slightly from 2000 to 2022, but generally maintains an upward trend year by year. To further predict the publication trend of sleep deprivation-induced cognitive dysfunction, a binomial function of the annual publication trend in this field from 2000 to 2022 was screened based on the correlation coefficient *R*^2^.^[34]^ After analysis, the binomial function *y* = 0.4434*x*^2^ −0.488*x* + 21.623 (*R*^2^ = 0.9801, *y* is the annual publication, *x* is the year), the inflection point has not yet appeared, which indicates that the papers in this field have grown rapidly in the past few years, and the global research on this field is still in the boom period, and the number of publications in the related field will maintain the continued upward trend in the subsequent years as the research continues to deepen. The number of citations in this field is also increasing year by year. With the rise in the heat of research related to sleep deprivation-induced cognitive dysfunction, the annual number of citations in this field is exploding from 2018 to 2021, and the slight decrease in the number of citations in the literature in 2022 may be related to the short dissemination time of the literature.

### 3.2. Country and regional analysis

The analysis of the obtained raw literature data into the visualization software shows that research in this field is mainly published by 73 countries or regions. The top 10 countries and regions in terms of number of publications are listed in Table 1, which also includes Centrality, Citation and Total link strength. The United States of America (USA) is the most published country with 922 publications in this field, accounting for 40.44% of the total number of publications in this field (n = 922, 40.44%), followed by China (The People’s Republic of China, PEOPLES R CHINA) (n = 189, 8.29%) and the United Kingdom (ENGLAND) (n = 169, 7.41%). The United States published more than 40% of the relevant literature published in the field, and it is clear that the United States published the largest number of papers with the highest number of citations and the strongest total link strength, indicating the strong influence of U.S. research in the field.

**Table 1:** Ranking of national publications of sleep deprivation related cognitive dysfunction in Web of Science Core Collection database between 2000 and 2022.

No.	Country	Centrality	Documents	Citation	Total link strength
1	USA	0.34	922	55,721	5546
2	PEOPLES R CHINA	0.00	189	3452	1675
3	ENGLAND	0.42	169	8461	1382
4	CANADA	0.04	142	7922	1392
5	AUSTRALIA	0.08	126	4780	1097
6	FRANCE	0.20	123	4323	1017
7	GERMANY	0.20	108	4948	631
8	ITALY	0.20	105	3706	705
9	NETHERLANDS	0.01	77	4278	879
10	BRAZIL	0.15	63	1608	323

**Figure 2.**
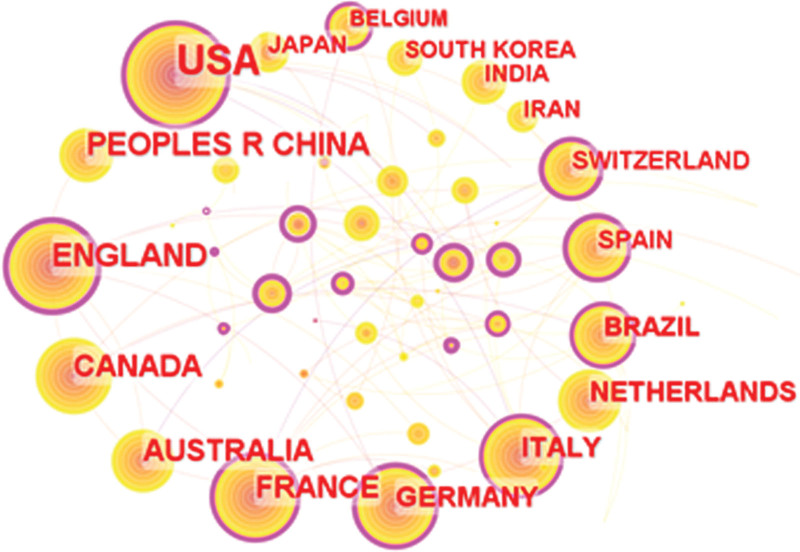
National visualization analysis atlas of literatures related to sleep deprivation related cognitive dysfunction between 2000 and 2022 in Web of Science Core Collection database.

The collaboration between different countries and regions is shown in Figure 2, where there are 73 nodes and 282 links. The size of the node represents the number of papers published in that country, and the red circle outside the node represents the number of papers published in the field by that country in recent years. The purple circle outside the node represents that the node has a high centrality, indicating that the country has an important position in the partnership. The top 5 countries and regions in centrality are UK (centrality = 0.42), USA (centrality = 0.34), France (FRANCE) (centrality = 0.20), Germany (GERMANY) (centrality = 0.20), and Italy (ITALY) (centrality = 0.20). As seen in Table 1 and Figure 2, the United States dominates research in the field of sleep deprivation-related cognitive dysfunction, far surpassing other countries in terms of number of papers; however, the United Kingdom has the highest centrality in terms of inter-country collaboration, indicating that the United Kingdom has good collaboration with several countries in field research.

### 3.3. Analysis of research organization

The analysis of the obtained raw literature data imported into the visualization software reveals that a total of 566 different organizations mainly published relevant literature in this field of research. Table 2 shows the top 10 research organizations with the highest number of publications, along with centrality, citations, and total link strength. The University of Pennsylvania (Univ Penn) had the highest number of publications with a total of 71 (3.11%), followed by Harvard University, Harvard (n = 54, 2.37%) and Stanford University, Stanford (n = 39, 1.71%).

**Table 2:** Ranking of research organization publications of sleep deprivation related cognitive dysfunction in Web of Science Core Collection database between 2000 and 2022.

No.	Institution	Documents	Centrality	Citation	Total link strength	Country
1	Univ Penn	71	0.16	7987	7097	USA
2	Harvard Univ	54	0.06	3679	4904	USA
3	Stanford Univ	39	0.06	2407	3425	USA
4	Harvard Med Sch	37	0.01	3909	899	USA
5	Univ Calif San Diego	36	0.08	6677	2548	USA
6	Washington State Univ	33	0.02	1385	1558	USA
7	Brigham & Women’s Hosp	31	0.02	3781	1367	USA
8	Walter Reed Army Inst Res	29	0.02	2338	2199	USA
9	Johns Hopkins Univ	28	0.05	3025	3238	USA
10	Univ Michigan	27	0.09	2288	1456	USA

Among the top 10 research organizations in terms of number of publications, the University of Pennsylvania (n = 7987) and the University of California, San Diego (UCSD) (n = 6677) had more than 5000 citations. The strongest total link was to the University of Pennsylvania at 7097. Johns Hopkins University published no more than 30 articles, but the institution had more than 3200 total citations, indicating that the institution is in the developmental stage of research but is making a significant impact in the field. All of the top 10 institutions in terms of number of publications are from the United States, which is consistent with the results of the analysis of countries and regions above.

**Figure 3.**
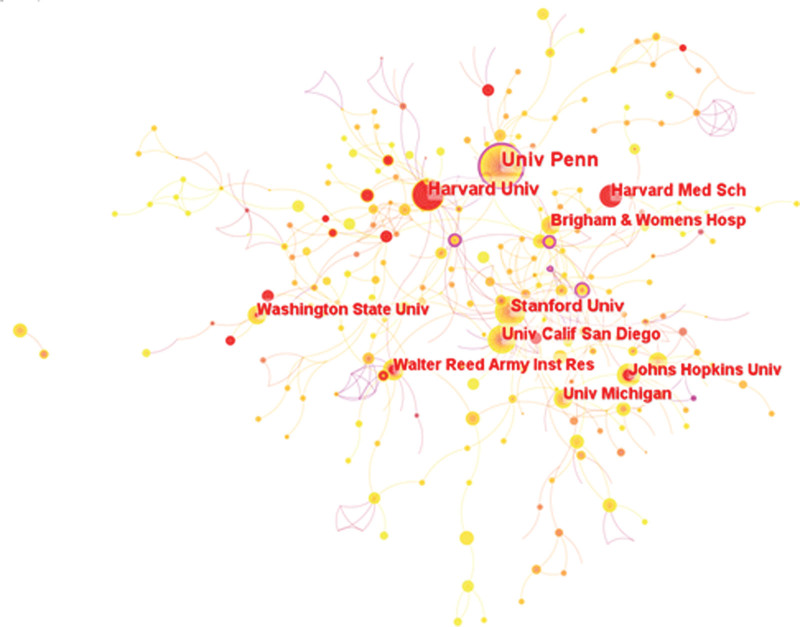
Research organization analysis atlas of literatures related to sleep deprivation related cognitive dysfunction between 2000 and 2022 in Web of Science Core Collection database Science Core Collection database.

The map of inter-institutional collaboration is shown in Figure 3, which includes a total of 566 nodes and 780 inter-node links. The size of the nodes represents the number of papers published by the institution, the red circles outside the nodes represent the number of papers published by the institution in the field in recent years, and the purple circles outside the nodes represent the institution with high centrality, indicating that the institution is in an important position in the collaboration map. Among them, the University of Pennsylvania has the highest centrality (n = 0.16), indicating that the institution is in a more important position in institutional collaboration and cooperates more closely with other institutions in the field.

### 3.4. Analysis of related journals

The analysis of the obtained raw literature data imported into the visualization software revealed that the main research in this field was published by a total of 714 journals on sleep deprivation-related cognitive dysfunction from 2000 to 2022. By analyzing the journals related to this field, we can identify the main influential journals in the specific research area. Table 3 lists the 10 journals with the highest number of published papers in 2022 and their impact factors. The top 3 journals are SLEEP (n = 138, IF = 6.313), JOURNAL OF SLEEP RESEARCH (n = 60, 5.296), and SLEEP MEDICINE (n = 48) (IF = 4.842). With the current impact factor assessment, SLEEP MEDICINE REVIEWS (IF = 11.401), SLEEP (IF = 6.313) and JOURNAL OF SLEEP RESEARCH (IF = 5.296) are the journals with high authority in this field.

**Table 3:** Ranking of journal publications of sleep deprivation related cognitive dysfunction in Web of Science Core Collection database between 2000 and 2022.

No.	Journals	Documents	Rate in total %	2022IF
1	SLEEP	138	6.05%	6.313
2	JOURNAL OF SLEEP RESEARCH	60	2.63%	5.296
3	SLEEP MEDICINE	48	2.11%	4.842
4	SLEEP MEDICINE REVIEWS	36	1.58%	11.401
5	PLOS ONE	32	1.40%	3.752
6	BEHAVIOURAL BRAIN RESEARCH	28	1.23%	3.352
7	SCIENTIFIC REPORTS	26	1.14%	4.996
8	CHRONOBIOLOGY INTERNATIONAL	23	1.01%	3.749
9	FRONTIERS IN NEUROSCIENCES	23	1.01%	5.152
10	JOURNAL OF CLINICAL SLEEP MEDICINE	22	0.96%	4.324

A co-cited journal is one in which two or more publications in the field of sleep deprivation-related cognitive dysfunction cite papers in the same journal. Table 4 shows the top 10 co-cited journals in the field. The top 3 co-cited journals were SLEEP (n = 1565), J SLEEP RES (n = 1102), and SLEEP MED REV (n = 894). The journal with the highest impact factor was NATURE (IF = 69.504). Analysis of the centrality of the co-cited journals showed that the centrality of all types of journals was less than 0.1, indicating that citation in the field is relatively average and no journals with significant centrality have been formed.

**Table 4:** Ranking of co-citation journal publications of sleep deprivation related cognitive dysfunction in Web of Science Core Collection database between 2000 and 2022.

No.	Journals	Documents	Centrality	2022IF (JCR)
1	SLEEP	1565	0.01	6.313
2	J SLEEP RES	1102	0.01	5.296
3	SLEEP MED REV	894	0.01	11.401
4	J NEUROSCI	860	0.02	6.709
5	SLEEP MED	781	0.03	4.842
6	PLOS ONE	750	0.01	3.752
7	P NATL ACAD SCI USA	698	0.01	12.779
8	SCIENCE	672	0.01	63.714
9	NATURE	602	0.01	69.504
10	BIOL PSYCHIAT	593	0.00	12.81

### 3.5. Author analysis

The analysis of the obtained raw literature data imported into the visualization software shows that the research in this field is mainly conducted by a total of 691 authors involved in 1503 relevant publications from 2000 to 2022. Table 5 shows the top 10 authors in terms of number of publications (in order of centrality if the number of publications is the same), their latest affiliation, number of author publications, centrality and H-index. Van dongen, Hans PA from Washington State University, Spokane, USA, had the highest number of publications (n = 22, 0.96%), followed by Balkin, Thomas Jf from the US Army (n = 14, 0.61%) and Balkin, Thomas Jf from the Dinges, David F (n = 13, 0.57%) from the University of Pennsylvania School of Medicine of Pennsylvania.

**Table 5:** Ranking of authors publications of sleep deprivation related cognitive dysfunction in Web of Science Core Collection database between 2000 and 2022.

No.	Author	Affiliation	Documents	H-index	Citation
1	Van dongen, Hans P A	Washington State University, Spokane, USA	22	44	1303
2	Balkin, Thomas J	United States Army	14	41	946
3	Dinges, David F	University of Pennsylvania Medicine	13	81	2277
4	Alzoubi, Karem H	University of Sharjah	12	39	602
5	Killgore, William D S	University of Arizona	11	48	1334
6	Sheibani, Vahid	Kerman University of Medical Sciences Lorestan University of Medical Sciences	10	28	345
7	Goel, Namni	Rush University	10	31	912
8	Ancoli-israel, Sonia	University of California System	9	97	1321
9	Gozal, David	University of Missouri Columbia	9	102	1703
10	Meerlo, Peter	University of Groningen	8	52	966

**Figure 4.**
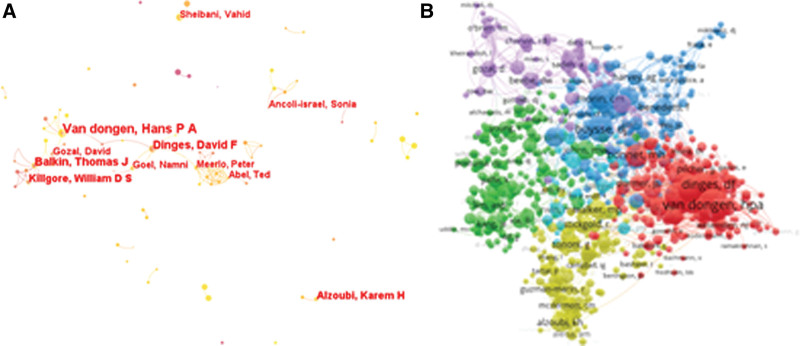
(A) Authors and collaborations in the field of cognitive impairment induced by sleep deprivation. (B) Co-cited authors in the field of cognitive impairment induced by sleep deprivation.

The collaboration between these authors is shown in Figure 4A, including a total of 748 nodes and 947 inter-node links. The size of the nodes of different authors represents the number of papers published by that author, the red circles outside the nodes represent the amount of publications in the field by that author in recent years, the purple circles outside the nodes represent the high centrality of that author, and the nodes with purple circles indicate that the author is in an important position in the collaboration among authors. Among all the authors, none of the nodes have a degree greater than 0.1, indicating that there is no leader in the field yet. Two authors’ articles are cited simultaneously in 1 literature, indicating a co-citation relationship between these 2 authors.^[35]^ Figure 4B shows the graph of co-cited authors in the relevant literature. After cluster analysis by VOSviewer, 5 clusters were identified with Van dongen, hpa (red), Lim, asp (green), Buysse,dj (blue), Alzoubi, Kh (yellow) and gozal,d (purple) as the core authors.

### 3.6. Keyword analysis

Keywords are a high level summary and condensation of the content of the literature, and their frequency of occurrence can reflect the research direction and hotspot of the research area.^[36]^ Keywords with only case differences, hyphen differences, abbreviations or similar intent will be combined, e.g. “ Sleep Deprivation,” “Insufficiencies, Sleep,” “ Sleep Fragmentation” will be combined into “ Sleep Deprivation.” By understanding the keywords shown in the main studies, we can explore the hot spots of current research and trends of future research.

**Figure 5.**
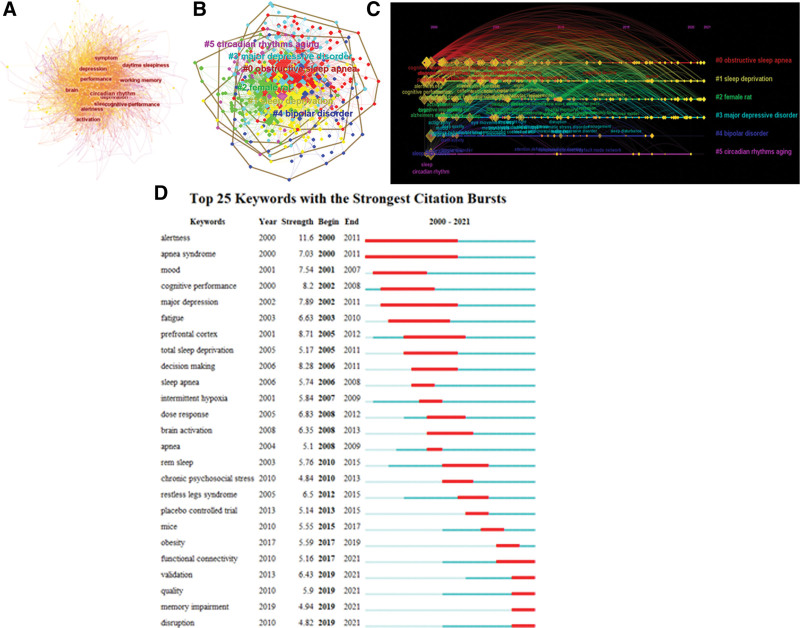
(A) Keywords collaborations in the field of cognitive impairment induced by sleep deprivation. (B) Keywords clustering analysis in the field of cognitive impairment induced by sleep deprivation. (C) Keywords time line spectrogram analysis in the field of cognitive impairment induced by sleep deprivation. (D) Top 25 keywords with the strongest citation bursts.

After screening and merging, a total of 646 relevant keywords were extracted, with 646 nodes and 2829 inter-node links in the keyword co-occurrence (Fig. 5A). Three keywords appeared more than 200 times: sleep deprivation “ Sleep Deprivation” (n = 650), deprivation “Deprivation” (n = 403), performance “Performance” (n = 325); 13 keywords appeared more than 100 times: Alzheimer’s Disease (n = 178), Cognitive Performance n = 177, Brain n = 150, Impairment n = 152, Memory n = 149, Circadian Rhythm n = 144, Disorder n = 143, Cognitive Function n = 132, Working Memory n = 130, Rem Sleep n = 104, Fatigue n = 103, Quality Of Life n = 101, Cognitive Impairment n = 100. 100. Keyword centrality above 0.1 indicates that the keyword is more likely to be a hot topic for research, with 1 keyword above 0.1, sleep deprivation (n = 0.11).

Cluster analysis identifies similar parts of keywords and combines them into a cluster that indicates the current state of research in the field. Keyword timeline spectrum analysis can analyze the timing and continuation of keyword appearances. Identifying keywords that change in frequency within a certain period of time can be used to reflect the hotspot phase of a research field, and combined with the research zone map of keywords, the evolution of hotspots in the research process of the field can be summarized, which has a certain guiding significance for the future research direction of the field. In Figure 5B, the modularity *Q* = 0.326 > 0.3 and the mean silhouette *S* value = 0.7062 > 0.7, indicating that the structure of this clustering association is significant and effective. In the keyword clustering plot (Fig. 5B) and the timeline profile (Fig. 5C), a total of 6 clusters were shown, its main themes are shown in Table 6, namely obstructive sleep apnea, sleep deprivation, female rats, major depression, bipolar disorder, and circadian rhythm aging. From the keyword clustering analysis and timeline spectrum analysis, it can be found that the clusters were studied from 2000 to 2022, and the research in this field was more concentrated before 2010, with larger keyword nodes shown in the figure; after 2010, it was more deeply studied within each branch, with smaller keyword nodes shown in the figure, and continued until now.

**Table 6:** Co-occurrence clustering information of key words related to sleep deprivation related cognitive dysfunction from 2000 to 2022 in Web of Science Core Collection database between 2000 and 2022.

Number	Documents	Contour	Year	Label
0	105	0.737	2005	Obstructive sleep apnea; sleep-disordered breathing; cognitive function; sleep deprivation; sleep apnea cognitive decline; clinical practice; sleep fragmentation; sleep problem; risk factor
1	97	0.661	2008	Sleep deprivation; cognitive performance; cognitive function; total sleep deprivation; sleep loss executive function; trait-like vulnerability; extended wakefulness; systematic review; sleep disorder
2	85	0.73	2010	Sleep deprivation; cognitive function; female rat; synaptic plasticity; sleep-deprived rat cognitive decline; systematic review; chronic sleep restriction; potential role; hippocampal vulnerability
3	74	0.675	2010	Sleep deprivation; sleep loss; cognitive performance; cognitive control; American academy sleep disturbance; sleep quality; systematic review; young adolescent; indirect effect
4	71	0.667	2011	Sleep disturbance; cognitive decline; sleep deprivation; sleep disorder; sleep fragmentation bipolar disorder; rem sleep; rem sleep deprivation; synergic nigral proapoptotic effect; rotenone model
5	63	0.678	2012	Sleep deprivation; sleep problem; insufficient sleep; systematic review; risk factor sleep quality; sleep disturbance; covid-19 pandemic; sleep disorder; cross-sectional study
6	59	0.806	2008	Sleep deprivation; bipolar disorder; functional connectivity, bipolar depression; resting state obstructive sleep apnea; systematic review; young men; differential effect; insufficient sleep
7	22	0.913	2003	Sleep deprivation; chronic sleep deprivation; deprivation-induced deficit; adult rat; sleep fragmentation young adult; sustained attention; habitual control; goal-directed control; chronic insomniac

Burst word detection is the digitization of the frequency of a specific topic during a research period, which can be used to study past research hotspots and predict potential future research directions in the field. The top 25 keywords with the strongest citation bursts in keyword citation burst detection are shown in Figure 5D. Alertness and apnea syndrome were the keywords with the longest bursts, with burst intensities of 11.6 and 7.03, respectively, from 2000 to 2011. the top 2 keywords with the strongest bursts were alertness and prefrontal The top 2 keywords with the highest burst intensity were Alertness and Prefrontal cortex, with strengths of 11.6 and 8.71, respectively, and bursts from 2000 to 2011 and 2005 to 2012. Other keywords with recent citation bursts include interference, memory impairment, quality, validation, and functional connectivity.

### 3.7. Analysis of highly cited articles

Analysis of the retrieved publications and co-cited references allowed the identification of current research hotspots in the field. Table 7 lists the 10 most cited papers published in the field between 2000 and 2022, with citation counts ranging from 636 to 1137. Table 8 lists the 10 most co-cited papers in the field between 1989 and 2022, with citations ranging from 135 to 209. The 4 papers cited and co-cited in both tables are the same and were published by Durmer, JS 2005, Thomas, M 2000, Van Dongen, HPA 2004 and Killgore, WDS 2010. They are from Georgia State University (GSU), the University of Texas at Austin (UT-Austin), Washington state university, Spokane, USA (Washington State University) and the University of Arizona (UA). All authors are from the United States. The conclusions are similar to the analysis between countries, which implies that the USA has a greater influence in this research area. The most cited paper was “Neurocognitive consequences of sleep deprivation,” published in 2005 by Durmer, JS, from Georgia State University in USA.

**Table 7:** Ranking of citation publications of sleep deprivation related cognitive dysfunction in Web of Science Core Collection database between 2000 and 2022.

No.	Title	First author	Journal	Citation	Publication year
1	Neurocognitive consequences of sleep deprivation	Durmer, JS	SEMINARS IN NEUROLOGY	1137	2005
2	A cognitive model of insomnia	Harvey, AG	BEHAVIOR RESEARCH AND THERAPY	927	2002
3	Diagnosis and management of childhood obstructive sleep apnea syndrome	Marcus, CL	DIAGNOSIS AND MANAGEMENT OF CHILDHOOD OBSTRUCTIVE SLEEP APNEASYNDROME	854	2012
4	Behavioral and physiological consequences of sleep restriction	Banks, S	JOURNAL OF CLINICAL SLEEP MEDICINE	827	2007
5	The association of sleep and pain: an update and a path forward	Finan, PH	JOURNAL OF PAIN	705	2013
6	Neural basis of alertness and cognitive performance impairments during Sleepiness. I. effects of 24 h of sleep deprivation on waking human regional brain activity	Thomas, M	JOURNAL OF SLEEP RESEARCH	702	2000
7	Sleep-disordered breathing, hypoxia, and risk of mild cognitive impairment and dementia in older women	Yaffe, K	JAMA-JOURNAL OF THE AMERICAN MEDICAL ASSOCIATION	675	2011
8	Effects of sleep deprivation on cognition	Killgore	HUMAN SLEEP AND COGNITION,PART I: BASIC RESEARCH	639	2010
9	Systematic interindividual differences in neurobehavioral impairment from sleep loss: evidence of trait-like differential vulnerability	Van Dongen	SLEEP	636	2004
10	Front cingulate dysfunction in depression: toward biomarkers of treatment response	Pizzagalli	NEUROPSYCHOPHARMACOLOGY	583	2011

**Table 8:** Ranking of Co-citation publications of sleep deprivation related cognitive dysfunction in Web of Science Core Collection database between 2000 and 2022.

No.	Title	First author	Journal	Citation	Publication year
1	The cumulative cost of additional wakefulness: dose-response effects on neurobehavioral functions and sleep physiology from chronic sleep restriction and total sleep deprivation	Van Dongen	SLEEP	209	2003
2	The Pittsburgh sleep quality index - a new instrument for psychiatric practice and research	BUYSSE, DJ	PSYCHIATRY RESEARCH	194	1989
3	A new method for measuring daytime sleepiness - the Epworth sleepiness scale	JOHNS, MW	SLEEP	173	1991
4	Neurocognitive consequences of sleep deprivation	Durmer, JS	SEMIN NEUROL	162	2005
5	Neural basis of alertness and cognitive performance impairments during sleepiness. i. effects of 24 h of sleep deprivation on waking human regional brain activity	Thomas, M	JOURNAL OF SLEEP RESEARCH	154	2000
6	Systematic interindividual differences in neurobehavioral impairment from sleep loss: evidence of trait-like differential vulnerability	Van Dongen, HPA	SLEEP	138	2004
7	A meta-analysis of the impact of short-term sleep deprivation on cognitive variables	Lim, J	PSYCHOLOGICAL BULLETIN	138	2010
8	Cumulative sleepiness, mood disturbance, and psychomotor vigilance performance decrements during a week of sleep restricted to 4-5 hours per night	Dinges, DF	SLEEP	135	1997
9	Effects of sleep deprivation on cognition	Killgore, WDS	PROG BRAIN RES	135	2010
10	Sleep drives metabolite clearance from the adult brain	Xie, LL	SCIENCE	135	2013

**Figure 6.**
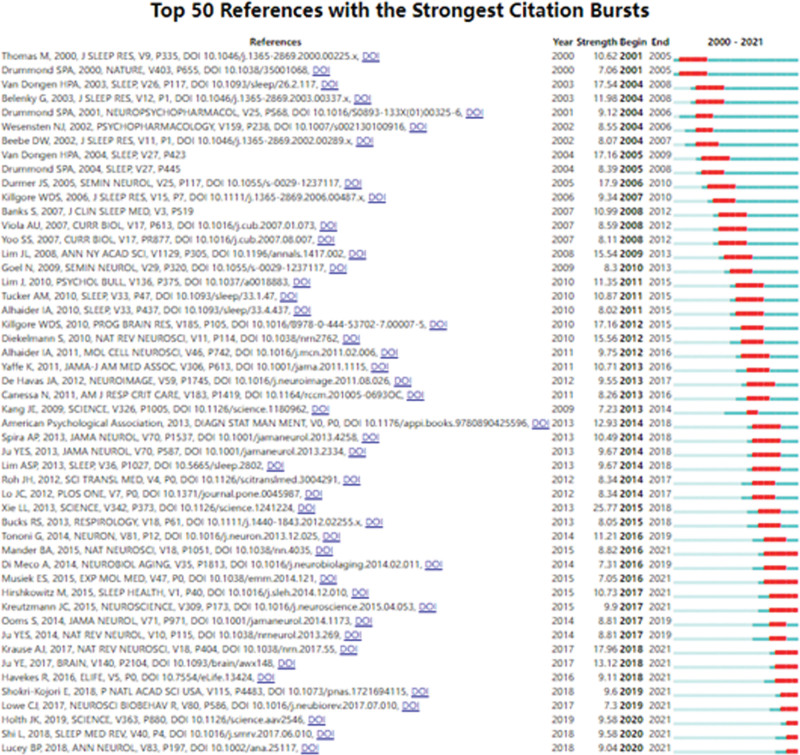
Top 50 publications with the strongest citation bursts.

Figure 6 lists the top 50 references with the strongest citation bursts from 2000 to 2022; the length of the blue line segment is the time frame of the study, and the part of the red line segment is the time period of the citation burst. The time period of the citation explosion is usually about 5 years, which may be related to the authors’ writing habits. Most authors cited articles within 5 years as references. The article with the strongest citation burst was LULU XIE’s study in 2014 with an intensity of 25.77 and a citation burst period of 2015 to 2018.^[37]^ Other references with citation burst intensity greater than 17 were published by Van Dongen HPA, Van Dongen HPA, Durmer JS, Killgore WDS, and Krause AJ. Most references focus on sleep deprivation-induced cognitive impairment, which usually manifests as nonspecific symptoms such as increased alertness and abnormal waking time, and is tolerated more differently in different individuals due to the specificity of the prefrontal cortex, and methods to eliminate this difference are still being explored. Recent highly cited references are Van Dongen 2003, BUYSSE, DJ 1989, JOHNS, MW 1991 and the Durmer, JS 2005 study. These studies mainly suggest that cognitive impairment due to sleep deprivation may be related to the quality and duration of sleep, the onset of sleep-related disorders, and the cumulative effect of sleep deprivation on cognitive impairment, so we need to explore better ways to gain insight into the causes of sleep deprivation in order to mitigate the development of severe cognitive impairment.

## 4. Discussion

As a field that examines research trends, direction, and guides future research, bibliometrics has significant value for scholars and researchers. In recent times, sleep deprivation-induced cognitive dysfunction has emerged as a prominent research focal point. The objective of this study is to present an overview of past and current research hotspots in this area and predict future research trends and directions.

Over the 20-year period analyzed, the number of publications in this research area has been steadily increasing with its peak expected in 2022. Additionally, the number of citations to these publications has been registering growth since 2000. An analysis of the publication growth curve suggests that the saturation point is yet to be achieved, indicating that the number of publications concerning this field will continue to rise. The majority of articles published in this area came from Western countries, with the United States leading the pack in both the number of articles published and cited, hinting at its strong influence in this area. China follows closely with the second-highest number of publications. The UK also has a strong presence in the field, contributing 7.41% of articles.

Among the 714 authors who published articles on sleep deprivation-induced cognitive impairment, the top 10 journals published 436 papers, representing 19.12% of all articles on the subject. SLEEP MEDICINE REVIEW published the most articles with 138 publications, representing 6.05%. It is evident that specialized journals in urology and oncology are more inclined towards publishing articles in this field. Interestingly, SLEEP MEDICINE REVIEW had the most impact factor (IF), indicating that sleep deprivation significantly impacts cognitive impairment. The top 10 co-cited journals are all listed in JCR Q1 and Q2 and include NATURE, SCIENCE, and P NATL ACAD SCI USA, with recent IFs of 69.504, 63.714, and 12.779, respectively. This highlights the relevance of high-impact factor journals when referencing sleep deprivation-induced cognitive impairment.

The analysis of authors and co-cited authors assists in identifying specialists with noteworthy influence in sleep deprivation-induced cognitive impairment research. Based on our analysis, Van Dongen, Hans P.A. was the most prolific author with 22 publications and the numerous collaborations. This study identified 5 clusters of co-cited authors with authors in the same cluster sharing collaborative relationships or similar research interests. These authors’ research primarily focused on neuroscience, clinical neurology, psychiatry, neuropharmacology, and the psychological aspects of sleep-related cognitive disorders.

## 5. Implications

The focus of research related to sleep deprivation-induced cognitive impairment can be explored from the co-occurring keywords. The 3 most common co-occurring keywords identified in our analysis include sleep deprivation, prefrontal cortex, and performance. Long-term or short-term sleep deprivation similarly may affect the normal physiological function of the prefrontal cortex, disrupting normal circadian rhythms and thus affecting normal daytime performance. The prefrontal cortex is a key region of the human brain related to higher cognitive functions, which plays a role in attention, emotion regulation, learning memory processes and thought reasoning^[12,38]^ and is particularly sensitive to sleep deprivation.^[39]^ After prolonged sleep deprivation, the overall metabolic level of the brain decreases, and the prefrontal cortex is one of the areas with the most pronounced decrease, while related functions of the prefrontal cortex such as working memory, emotion regulation, attention maintenance The prefrontal cortex is also vulnerable to sleep deprivation.^[40–43]^ Current research suggests that sleep deprivation adversely affects general lower-order cognitive functions,^[44,45]^ but the effects on higher-level cognitive abilities - executive functions - and the underlying mechanisms behind them are unclear.^[46,47]^ Executive functions primarily include working memory, inhibitory control, and cognitive flexibility.^[48]^ There is substantial empirical evidence that sleep deprivation affects the neural basis of executive functioning, suggesting that the prefrontal cortex plays a central role in the performance of executive functions. For example, studies have shown a significant decrease in inhibition efficiency after sleep deprivation, with decreased activation in ventral prefrontal cortex, anterior prefrontal cortex, and right anterior insula.^[49]^ In a study using near-infrared spectroscopy, it was found that those who did not receive sufficient sleep under acute sleep deprivation conditions lacked bilateral frontal activation during working memory processing and that the level of frontal activation was significantly reduced in sleep-deprived individuals.^[50]^ In addition, Nakashima et al^[51]^ used fMRI techniques to investigate the neural basis of the effects of sleep deprivation on cognitive flexibility and found that complete sleep deprivation activated regions of the frontal and parietal lobes as well as the cingulate gyrus. For patients who may produce cognitive impairment, better social support may relieve patients’ symptoms to a certain extent and slow down the progress of the disease.^[52]^ A more comfortable environment may reduce the discomfort of patients and promote the recovery of the disease. However, so far, there is a lack of a systematic overview of the hotspots and future research aspects of sleep deprivation-induced cognitive impairment, and further research in this area is needed. Relevant health professionals should also take corresponding preventive measures to improve the awareness of sleep deprivation in cognitive impairment, http://links.lww.com/MD/J878.

## 6. Limitations

At the time of research in this field, the field has gradually formed a certain development direction, and the guiding effect of this study on this field has been weakened to a certain extent. The same research should be carried out earlier to guide the research direction at the early stage of development.^[53]^ Our analysis of this field is still at the theoretical level to a certain extent, and we need more research that can be translated into visual results.^[54]^ For the analysis of literature, this paper studies this field from a macro perspective, and subsequent research can be carried out according to the specific research situation in different regions.^[55]^ Due to the differences in policies and actual conditions in different countries and regions, different research directions in different localities may show different developments in line with local conditions. When conducting research, local policies and actual conditions should also be considered.^[56]^

## 7. Conclusion

This study is a bibliometric analysis of the impact of sleep deprivation on cognitive function from 2000 to 2022. The study utilizes literature collected from the Web of Science Core Collection database to gain insight into research trends and directions in the field. The number of publications has steadily increased over the 23-year study period, and based on publication trends, it is expected to continue to rise in the future. The United States is a dominant force in sleep deprivation research, with influential scholars and institutions leading the field.

Keyword co-occurrence analysis revealed that Sleep Deprivation, Prefrontal Cortex, and Performance are the most significant keywords in the field, suggesting that sleep deprivation, whether short-term or long-term, can profoundly affect prefrontal cortical function and disrupt circadian rhythm, impacting daytime performance. As a potential therapeutic approach, improving sleep quality could help reduce cognitive impairment.

According to the conclusions found in the literature, early clinical intervention to improve sleep quality could successfully reduce the occurrence of subsequent cognitive impairment, although further randomized controlled trials are necessary to validate these results. In addition, personalized intervention measures should be tailored to individual patients according to their specific environments.

The prevalence of cognitive impairment during the epidemic era can be influenced by local prevention and control measures. While the post-pandemic era may still affect cognitive function, it is expected to improve over time.

## Acknowledgments

We appreciate the great support from Dr Li Rong from the Department of Plastic and Aesthetic Surgery, The First Hospital of Jilin University.

## Author contributions

**Conceptualization:** Kai Yu, Peng Li.

**Data curation:** Kai Yu, Fan Bu, Yuanzhi Guo.

**Formal analysis:** Kai Yu, Fan Bu, Yaqi Duan, Ji Lu.

**Funding acquisition:** Fan Bu, Yaqi Duan, Ji Lu, Peng Li.

**Investigation:** Yaqi Duan, Lei Hao, Peng Li.

**Methodology:** Lei Hao, Peng Li.

**Project administration:** Rui Hu, Lei Hao, Peng Li.

**Resources:** Kai Yu, Rui Hu.

**Software:** Kai Yu.

## Supplementary Material
