# Knowledge structure and emerging trends of cognitive impairment induced by sleep deprivation: A bibliometric analysis based on CiteSpace and VOSviewer from 2000 to 2022: Erratum

**DOI:** 10.1097/MD.0000000000037557

**Published:** 2024-03-15

In the article “Knowledge structure and emerging trends of cognitive impairment induced by sleep deprivation: A bibliometric analysis based on CiteSpace and VOSviewer from 2000 to 2022”^1^, which appears in Volume 102, Issue 40 of *Medicine* the correct corresponding author information is “Peng Li, The First Affiliated Hospital of Inner Mongolia Medical University, Inner Mongolia Autonomous Region, China (e-mail: hlp8079@163.com).”
