# Supplementary figures and images for: Knowledge structure and emerging trends of cognitive impairment induced by sleep deprivation: A bibliometric analysis based on CiteSpace and VOSviewer from 2000 to 2022
Source: Medicine (Baltimore). 2023 Oct 6;102(40):e34776. doi: 10.1097/MD.0000000000034776 (PMC10552981; doi:10.1097/MD.0000000000034776)

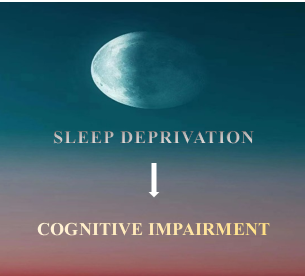

Supplement: Supplementary file 1 [file medi-102-e34776-s001.jpg]
